# Effects of Endogenous Signals and *Fusarium oxysporum* on the Mechanism Regulating Genistein Synthesis and Accumulation in Yellow Lupine and Their Impact on Plant Cell Cytoskeleton

**DOI:** 10.3390/molecules190913392

**Published:** 2014-08-29

**Authors:** Magda Formela, Sławomir Samardakiewicz, Łukasz Marczak, Witold Nowak, Dorota Narożna, Waldemar Bednarski, Anna Kasprowicz-Maluśki, Iwona Morkunas

**Affiliations:** 1 Department of Plant Physiology, Poznań University of Life Sciences, Wołyńska 35, Poznań 60-637, Poland; E-Mail: formelamagda@o2.pl; 2 Laboratory of Electron and Confocal Microscopy, Faculty of Biology, Adam Mickiewicz University, Umultowska 89, Poznań 60-614, Poland; E-Mail: sas@amu.edu.pl; 3 Institute of Bioorganic Chemistry, Polish Academy of Sciences, Z. Noskowskiego 12/14, Poznań 61-704, Poland; E-Mail: lukasmar@ibch.poznan.pl; 4 Laboratory of Molecular Biology Techniques, Faculty of Biology, Adam Mickiewicz University, Umultowska 89, Poznań 60-614, Poland; E-Mail: nowak@amu.edu.pl; 5 Department of Biochemistry and Biotechnology, Poznań University of Life Sciences, Dojazd 11, Poznań 60-632, Poland; E-Mail: dorna@o2.pl; 6 Institute of Molecular Physics, Polish Academy of Sciences, Smoluchowskiego 17, Poznań 60-179, Poland; E-Mail: Waldemar.Bednarski@ifmpan.poznan.pl; 7 Department of Molecular and Cellular Biology, Faculty of Biology, Adam Mickiewicz University, Umultowska 89, Poznań 60-614, Poland; E-Mail: ania.k.kasprowicz@gmail.com

**Keywords:** genistein, sugars, actin and tubulin cytoskeletons, free radicals, *Lupinus luteus*, *Fusarium oxysporum*

## Abstract

The aim of the study was to examine cross-talk interactions of soluble sugars (sucrose, glucose and fructose) and infection caused by *Fusarium oxysporum* f.sp. *lupini* on the synthesis of genistein in embryo axes of *Lupinus luteus* L.cv. Juno. Genistein is a free aglycone, highly reactive and with the potential to inhibit fungal infection and development of plant diseases. As signal molecules, sugars strongly stimulated accumulation of isoflavones, including genistein, and the expression of the isoflavonoid biosynthetic genes. Infection significantly enhanced the synthesis of genistein and other isoflavone aglycones in cells of embryo axes of yellow lupine with high endogenous sugar levels. The activity of β-glucosidase, the enzyme that releases free aglycones from their glucoside bindings, was higher in the infected tissues than in the control ones. At the same time, a very strong generation of the superoxide anion radical was observed in tissues with high sugar contents already in the initial stage of infection. During later stages after inoculation, a strong generation of semiquinone radicals was observed, which level was relatively higher in tissues deficient in sugars than in those with high sugar levels. Observations of actin and tubulin cytoskeletons in cells of infected embryo axes cultured on the medium with sucrose, as well as the medium without sugar, showed significant differences in their organization.

## 1. Introduction

Genistein (C_15_H_10_O_5_, 5,7,4'-trihydroxyisoflavone) is a fascinating molecule, attracting the attention of researchers due to its broad spectrum of its biological activity not only in plants, but also in the human organism. Plant-origin genistein in animals and human body cells acts as an anticancer, antibacterial and spasmolytic substance, but it also has antioxidant and hypotensive effects [1]. Moreover, genistein as a natural isoflavonoid phytoestrogen, is a strong inhibitor of protein tyrosine kinases [2]. Increased or aberrant expression of tyrosine kinases impact on tumor development and progression. This bioactive aglycone can also affect metabolism, *i.e.*, the kinetics of insulin binding to cell membranes [3], leptin secretion–an important factor regulating the energetic status of the whole organism [4].

In plant cells the physiological function of genistein has been established, it is a free aglycone of isoflavonoid origin capable of inhibiting the development of infections and diseases caused by pathogenic fungi [5]. Genistein may function as a phytoalexin due to its antimicrobial and fungistatic activity [6,7,8]. Results of *in vitro* tests showed also that genistein is a strong inhibitor of growth of a pathogenic fungus [9]. Additionally, it serves as a signalling compound that initiates the nodulation process in the legume-rhizobia symbiosis [10,11]. Plants from the family Leguminosae are rich sources of genistein [12,13]. It was shown that genistein is found in lupine (*Lupinus luteus*, *Lupinus albus*) [9,14,15], soy (*Glycine* sp.) [16,17], clover (*Trifolium* sp.) [18,19], in lucerne (*Medicago sativa*) [20,21] and in kudzu roots (*Pueraria lobata*) [22]. Genistein can be also secreted from roots of legumes [13]. Elucidation of the biosynthesis of numerous phytoalexins, including genistein, has facilitated the use of molecular biology tools in the exploration of the genes encoding enzymes of their synthesis pathways and their regulators [23]. 

Genetic modifications on the biosynthesis of phytoalexins was investigated in the context of plant resistance to pathogens. The effect of sucrose was demonstrated to improve the production of secondary metabolites, including flavonoids in plants [24,25]. Recent literature reports have documented both the essential role of sucrose as a donor of carbon skeletons for the metabolism of phenylpropanoid metabolism and that of the signalling molecules up-regulating expression of biosynthesis of phenylpropanoid [26,27,28,29]. Apart from sucrose, also glucose and fructose are recognized as signalling molecules in plants [30,31]. Sugar signals may contribute to immune responses against pathogens and probably function as priming molecules against pathogens [32]. The novel concept of “sweet priming” predicts specific key roles for saccharides in perceiving, mediating and counteracting both biotic and abiotic stresses.

The aim of the present study was to examine effects of sucrose and monosaccharides (glucose and fructose) as endogenous signals, and a hemibiotrophic fungus *Fusarium oxysporum* f.sp. *lupini* on the mechanism regulating genistein synthesis and its accumulation in embryo axes of *Lupinus luteus* L. cv. Juno. Therefore, apart from the estimation of levels of genistein and other isoflavone free aglycones, the expression of genes encoding phenylalanine ammonia-lyase (PAL), chalcone synthase (CHS), chalcone isomerase (CHI) and isoflavone synthase (IFS) was analyzed in the non-infected and *F. oxysporum*-infected embryo axes. Within this study we also analyzed changes in the activity of β-glucosidase, an enzyme which releases free aglycones from their glucoside bindings. At the exogenous addition of sucrose, glucose or fructose the endogenous levels of these sugars were determined in tissues. Moreover, generation of free radicals was estimated in view of the varied level of sucrose and monosaccharides, which may be incorporated in the defence responses of embryo axes, for example stimulation of phytoalexin synthesis or sealing of the cell walls. It was particularly interesting and important to examine post-infection changes in the actin and tubulin cytoskeleton cells of embryo axes of yellow lupine at varied levels of sucrose.

Available literature does not provide information regarding the importance of monosaccharides, *i.e.*, glucose and fructose, for the mechanism regulating genistein synthesis, generation of the superoxide anion radical and semiquinone radicals in plant response to infection. It should be stressed that the aforementioned aspects of the research, as well as monitoring of post-infection changes in actin and tubulin cytoskeletons in cells of yellow lupine embryo axes with different levels of sucrose after inoculation by *F. oxysporum*, are novel problems. 

## 2. Results and Discussion

### 2.1. The Effect of Sucrose, Glucose and Fructose on Accumulation of Genistein and Other Isoflavones in Embryo Axes Infected with F. oxysporum

**Figure 1:**
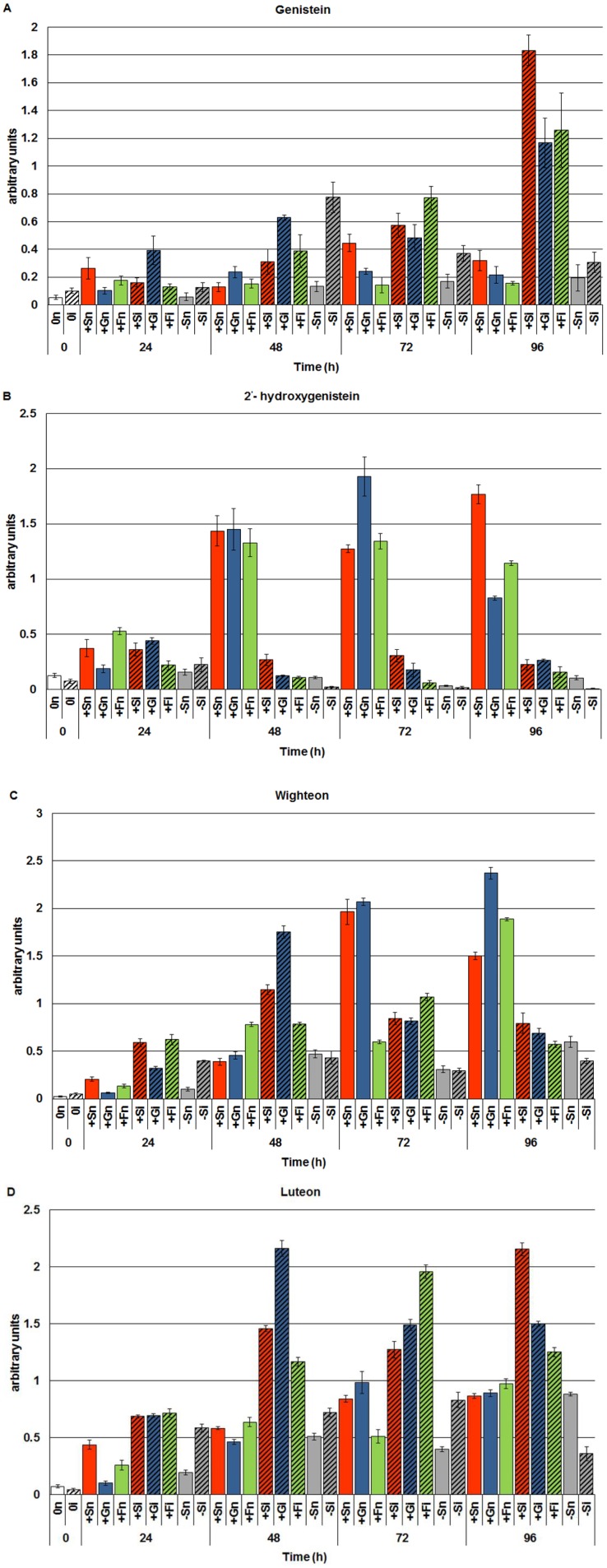
The effect of sucrose, glucose and fructose on accumulation of genistein (**A**) and other isoflavones, *i.e.*, 2'-hydroxygenistein (**B**), wighteone (**C**) and luteone (**D**), in *in vitro* cultured embryo axes of *Lupinus luteus* L. cv. Juno infected with *Fusarium oxysporum* f.sp. *lupine*. Statistical significance of differences between the average values of each pairs for accumulation of genistein and other isoflavones. Statistically significant differences (*p*-value) was assumed at *p* < 0.05.

Exogenous addition of sucrose, glucose or fructose to the medium as a rule caused higher concentrations of genistein and other isoflavones in non-inoculated embryo axes of yellow lupine cv. Juno (+Sn, +Gn and +Fn) than in non-inoculated axes cultured at carbohydrate deficit (−Sn) (Figure 1A). However, the level of genistein in these tissues was higher in axes cultured *in vitro* on the medium with sucrose (+Sn) than in axes cultured with monosaccharides (+Gn or +Fn). In turn, infection of embryo axes with a hemibiotrophic fungus *F. oxysporum* considerably enhanced the accumulation of genistein. Already at 24 h after inoculation the level of genistein was 4 times greater in embryo axes cultured on the medium with glucose (+Gi) than in non-inoculated axes (+Gn). Moreover, post-infection accumulation of genistein was also found at successive time points after inoculation in axes cultured on the medium with sucrose and monosaccharides, *i.e.*, glucose and fructose. However, we need to focus particularly on the very strong accumulation of genistein in these tissues at 96 h after inoculation. Moreover, the post-infection accumulation of genistein was also observed in axes inoculated with *F. oxysporum* cultured under carbohydrate deficit. The highest level of this free aglycone in these tissues was recorded at 48 h after inoculation, while at successive time points this level was much lower than in inoculated tissues with high levels of sugars (+Si, +Gi and +Fi). It is of interest that starting from 48 h of culture the level of 2'-hydroxygenistein was many times higher in non-inoculated axes cultured on the medium with sucrose, glucose or fructose (+Sn, +Gn or +Fn) than in the other experimental variants (−Sn, −Si, +Si, +Gi, +Fi) (Figure 1B). 

Infection caused a very strong reduction of the 2'-hydroxygenistein level in all inoculated embryo axes, occasionally so substantial that it reached the detection limit for this metabolite. As a result of inoculation with *F. oxysporum*, concentrations of another free aglycone, which is a prenylated derivative of 2'-hydroxygenistein, *i.e.*, luteone, increased many times in inoculated embryo axes with high levels of sugars (+Si, +Gi and +Fi) (Figure 1D). Moreover, in the period from 24 to 72 h after inoculation the level of luteone was higher in inoculated axes with carbohydrate deficit (−Si) than in non-inoculated ones (−Sn). In turn, the level of wighteone, a prenylated derivative of genistein, decreased considerably at 72 and 96 h after inoculation in axes with high levels of sucrose, glucose and fructose, while the concentration of genistein at these time points after inoculation was increased (Figure 1C).

### 2.2. The Effect of Sucrose, Glucose and Fructose on Expression Levels of Genistein Biosynthesis Pathway Genes in Response to Infection with F. oxysporum

**Figure 2:**
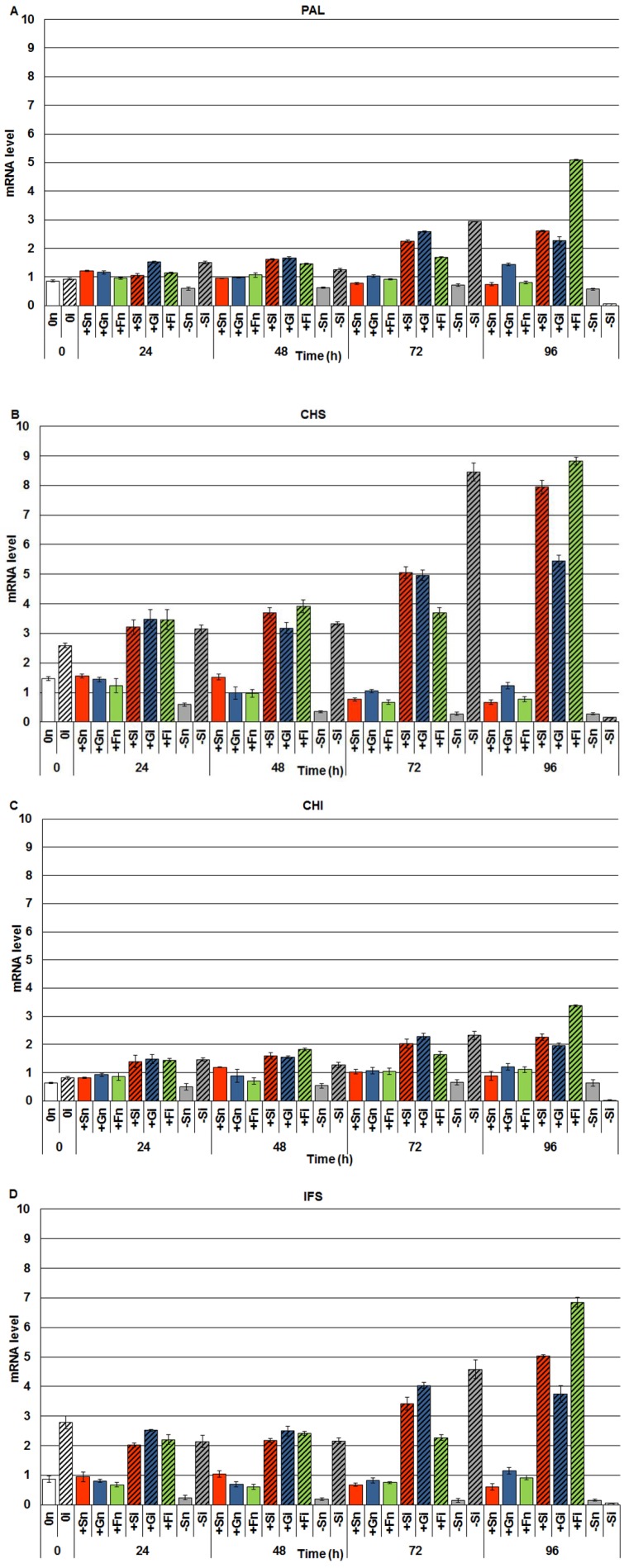
The effect of sucrose, glucose and fructose on expression levels of isoflavone biosynthetic pathway genes, *i.e.*, phenylalanine ammonia-lyase (PAL) (**A**), chalcone synthase (CHS) (**B**), chalcone isomerase (CHI) (**C**) and isoflavone synthase (IFS) (**D**) in *in vitro* cultured embryo axes of *L. luteus* infected with *F. oxysporum.* Statistical significance of differences between the average values of each pairs for expression levels of isoflavone biosynthetic pathway genes. Statistically significant differences (*p*-value) was assumed at *p* < 0.05.

Real-time PCR analyses of the level of mRNA encoding enzymes involved in the synthesis of isoflavones, including genistein, revealed post-infection accumulation of mRNA in embryo axes of yellow lupine cv. Juno (Figure 2). In embryo axes infected with *F. oxysporum* cultured *in vitro* on the medium with sucrose, glucose and fructose (+Si, +Gi, +Fi) the level of mRNA for PAL, CHS, CHI and IFS was higher than in non-infected axes (+Sn, +Gn, +Fn) in the period from 0 to 96 h. Moreover, up to 72 h after inoculation in inoculated axes with a sugar deficit (−Si) a higher level of mRNA encoding PAL, CHS, CHI and IFS was observed post infection in relation to non-inoculated axes (−Sn). At the next time point after inoculation, *i.e.*, at 96 h in infected axes cultured at carbohydrate deficit (−Si) a very strong reduction was recorded in the mRNA level, while at 96 h in infected axes with a high level of carbohydrates (+Si, +Gi and +Fi) the level of mRNA encoding the above mentioned enzymes was the highest. It needs to be stressed that a very high post-infection level of mRNA was recorded for enzymes of the specific isoflavone synthesis pathway, *i.e.*, CHS and IFS (Figure 2B,D) in embryo axes inoculated with *F. oxysporum*, being much higher than for PAL and CHI (Figure 2A,C). 

### 2.3. The Effect of Exogenous Sucrose, Glucose and Fructose on Endogenous Levels of Soluble Sugars and Their Changes in Response to Infection with F. oxysporum 

**Figure 3:**
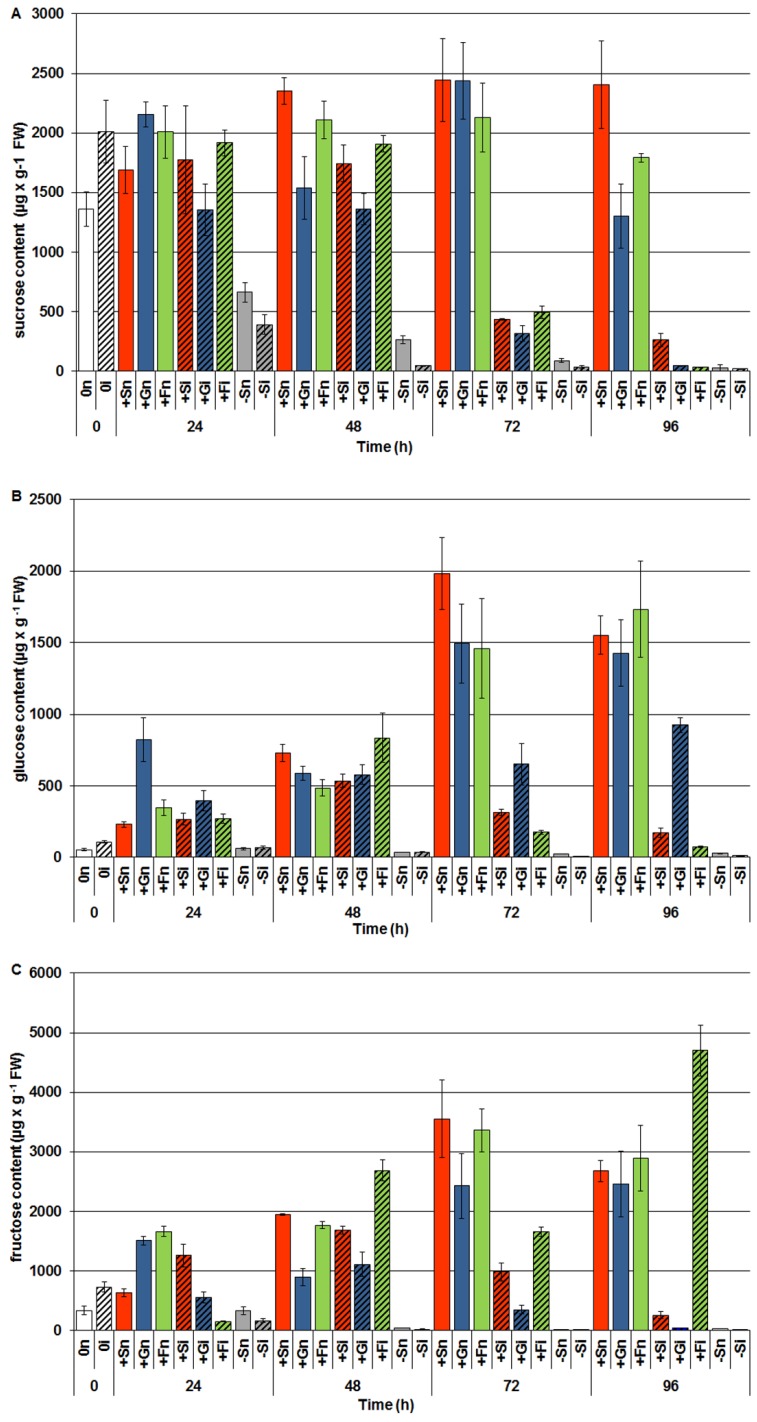
The effect of sucrose, glucose and fructose on the endogenous level of soluble sugars *i.e.*, sucrose (**A**), glucose (**B**), fructose (**C**) in *in vitro* cultured embryo axes of *L. luteus* and their changes in response to infection with *F. oxysporum.* Statistical significance of differences between the average values of each pairs for endogenous level of sugars. Statistically significant differences (*p*-value) was assumed at *p* < 0.05.

GC-MS analyses of the carbohydrate levels showed that after exogenous administration of sucrose, glucose and fructose the endogenous level of these sugars increased strongly in embryo axes of yellow lupine cv. Juno (Figure 3). Levels of sucrose and monosaccharides (glucose and fructose) in embryo axes of yellow lupine cultured *in vitro* with an addition of sucrose (+Sn), glucose (+Gn) and fructose (+Fn) was much higher than in embryo axes cultured at carbohydrate deficit (−Sn). A very strong endogenous decrease in the levels of sucrose (Figure 3A) and monosaccharides (glucose and fructose) (Figure 3B,C) was observed at 72 h after infection in embryo axes cultured in the presence of these carbohydrates. At 96 h after infection the level of sucrose decreased even further (Figure 3A), while the content of fructose in +Fi tissues increased considerably and was higher than in the other experimental variants (Figure 3C). 

What is more, already starting from 24 h after infection in embryo axes with carbohydrate deficit (−Si) the level of sugars decreased and it was lower than in non-inoculated embryo axes (−Sn) at all-time points. The level of carbohydrates in −Si and −Sn tissues was very low in relation to the other experimental variants. 

### 2.4. The Effect of Sucrose, Glucose and Fructose on β-Glucosidase Activity in Response to Infection with F. oxysporum

**Figure 4:**
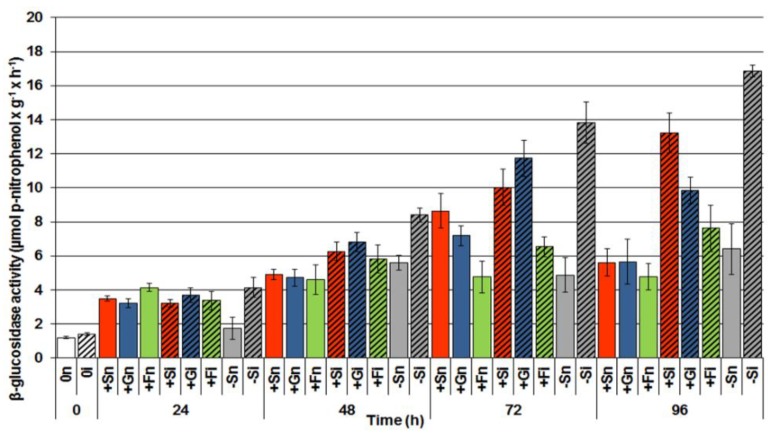
The effect of sucrose, glucose and fructose on β-glucosidase activity in *in vitro* cultured embryo axes of *L. luteus* infected with *F. oxysporum*. Statistical significance of differences between the average values of each pairs for β-glucosidase activity. Statistically significant differences (*p*-value) was assumed at *p* < 0.05.

Analyses of β-glucosidase activity in the period from 0 to 96 h of culture showed a post-infection increase in the activity of this enzyme in inoculated embryo axes both cultured in the presence of sucrose, glucose and fructose, and under carbohydrate deficit (Figure 4). However, the activity of this enzyme was higher in embryo axes infected with *F. oxysporum* cultured at carbohydrate deficit (−Si) than in the infected embryo axes cultured in the presence of sugars, *i.e.*, (+Si, +Gi, +Fi). The highest activity of β-glycosidase was recorded at 96 h after inoculation.

### 2.5. The Effect of Sucrose, Glucose and Fructose on Superoxide Anion Radical (O_2_^•−^) Generation in Response to Infection with F. oxysporum

**Figure 5:**
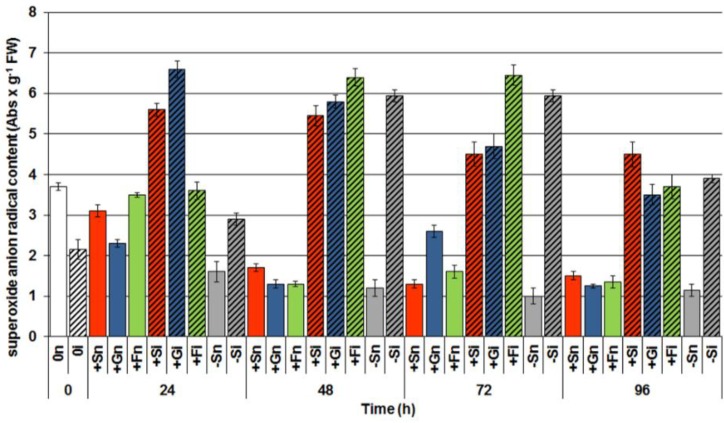
The effect of sucrose, glucose and fructose on superoxide anion (O_2_^•−^) generation in *in vitro* cultured embryo axes of *L. luteus* infected with *F. oxysporum*. Statistical significance of differences between the average values of each pairs for superoxide anion radical generation. Statistically significant differences (*p*-value) was assumed at *p* < 0.05.

In the period from 24 to 96 h after inoculation a strong generation of the superoxide anion radical was observed in embryo axes infected with *F. oxysporum* (Figure 5).

At 24 h after inoculation a particularly interesting finding was connected with the high level of O_2_^•−^ generation in infected embryo axes with a high level of sugars (+Si, +Gi and +Fi), with this level being considerably higher than in embryo axes with the deficit (−Si). At successive time points after inoculation intensity of O_2_^•−^ generation in −Si embryo axes was similarly as high as in the infected embryo axes with a high endogenous level of carbohydrates (+Si, +Gi and +Fi).

### 2.6. The Effect of Sucrose, Glucose and Fructose on Semiquinone Radical Generation in Response to Infection with F. oxysporum

**Figure 6:**
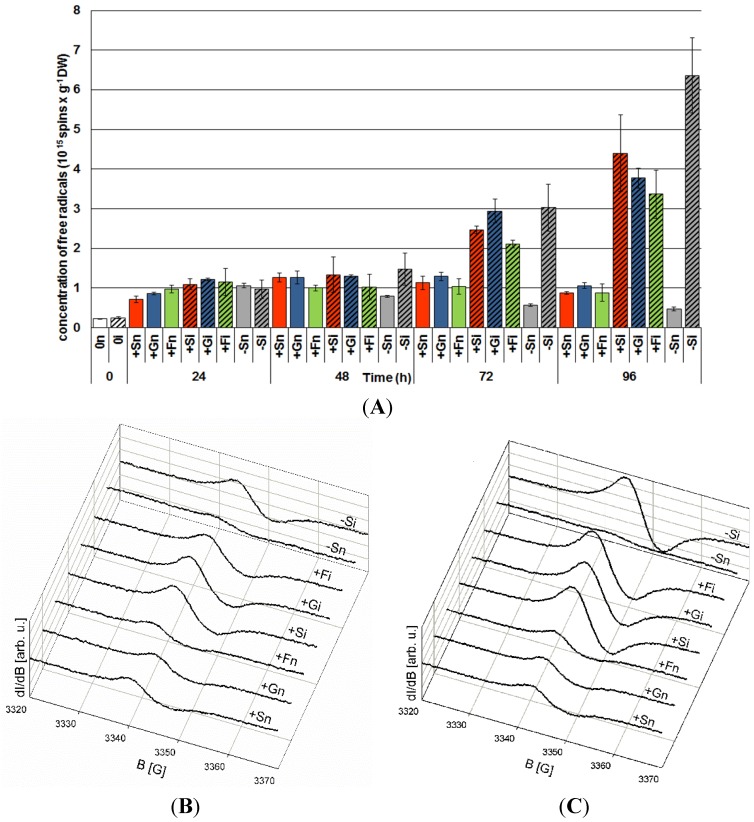
The effect of sucrose, glucose and fructose on semiquinone radical concentration in *in vitro* cultured embryo axes of *L. luteus* infected with *F. oxysporum* (**A**) and typical wide-scan EPR spectra at 72 h (**B**) and 96 h (**C**). Statistical significance of differences between the average values of each pairs for semiquinone radical concentration. Statistically significant differences (*p*-value) was assumed at *p* < 0.05.

Starting from 48 h culture the level of generation of semiquinone radicals with the Landé g factor $g_{\left|\right|}=2.0036(0.005)$ $g_{⊥}=2.0053(0.003)$ was higher in embryo axes with high endogenous levels of sucrose, glucose and fructose than in those with sugar deficits (Figure 6). At that time point it was observed that infection with *F. oxysporum* resulted in enhanced generation of these radicals in infected embryo axes with a carbohydrate deficit (−Si) in relation to those non-infected (−Sn). At further time points after inoculation with *F. oxysporum*, *i.e.*, at 72 and 96 h, concentrations of these radicals increased considerably, both in −Si and +Si, +Gi and +Fi embryo axes and it was much greater than in non-infected embryo axes (−Sn, +Sn, +Gn, +Fn) (Figure 6A). However, at 96 h after inoculation the concentration of these radicals was the highest in embryo axes with carbohydrate deficit (−Si), being higher than in infected embryo axes with a high endogenous carbohydrate level.

### 2.7. The Effect of Sucrose on Actin and Tubulin Cytoskeleton Organization in Response to Infection with F. oxysporum

**Figure 7:**
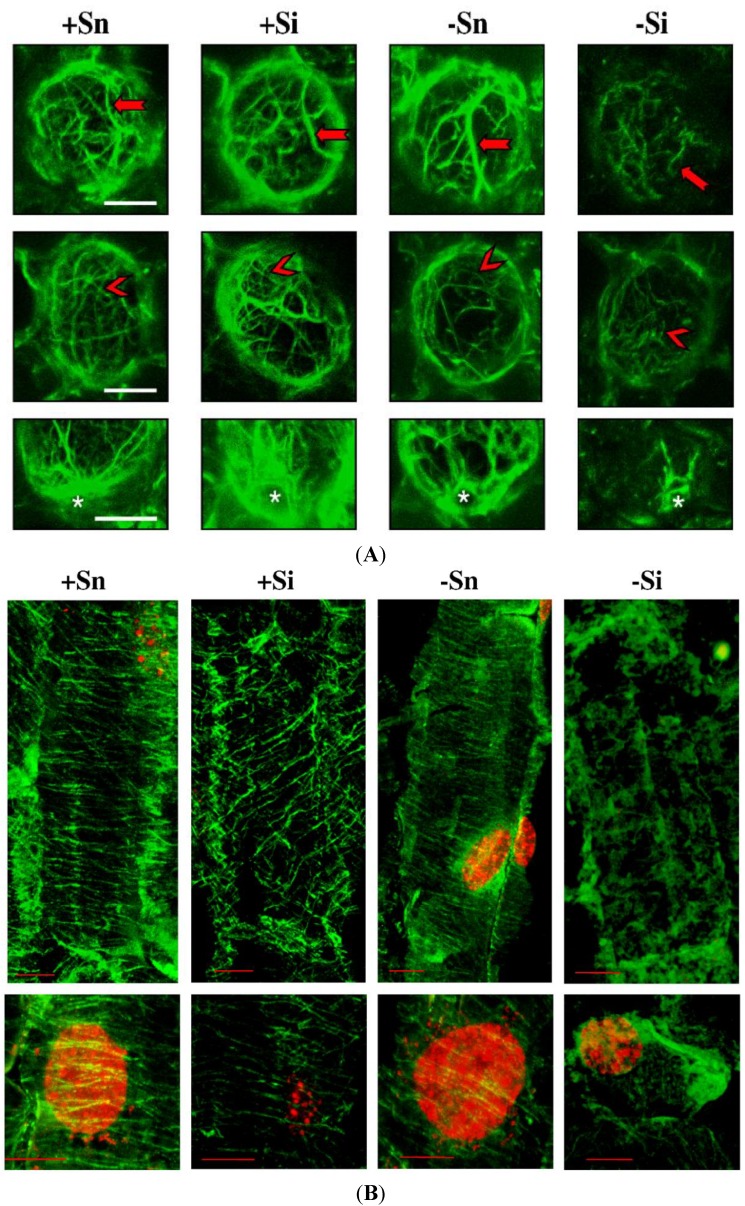
The effect of sucrose and *F. oxysporum* on actin (**A**) (scale = 20 µm) and tubulin (**B**) (green fluorescence; scale = 10 µm) cytoskeleton organization *in vitro* cultured embryo axes of *L. luteus*. The red arrow indicates actin cables, red arrowhead-meshwork of microfilaments and white star-actin cytoskeleton around the nucleus.

The actin cytoskeleton in cells of non-inoculated axes with a high endogenous level of sucrose (+Sn) was formed of long and thick actin cables surrounding the cells and their branches, creating a dense actin meshwork extending in various directions (Figure 7A). 

**Figure 8:**
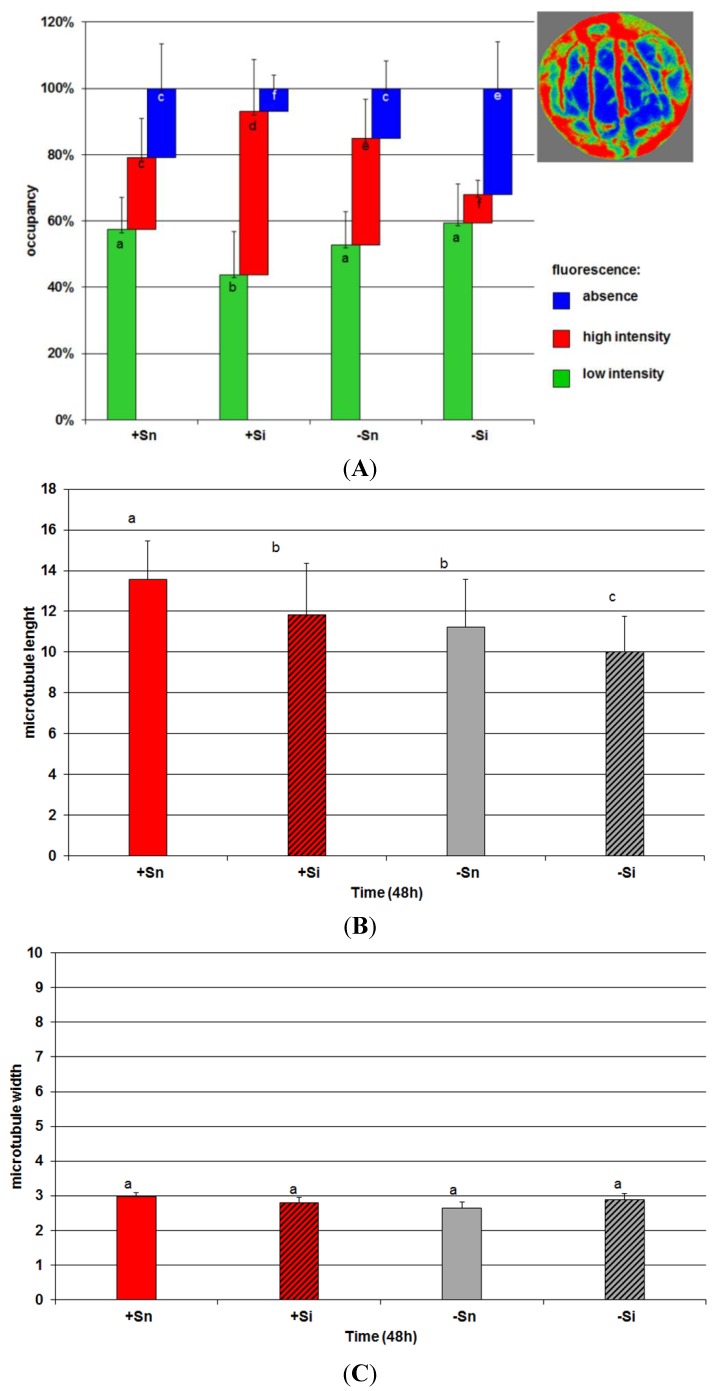
(**A**) The effect of sucrose and *F. oxysporum* on actin cytoskeleton density in cells of embryo axes of *L. luteus*. To estimate the density of microfilaments (MFs), the occupancy (%) of the pixel areas of AlexaFluor signals from segmented images (example image in legend) of cell was calculated. The red color represents areas of high fluorescence intensity corresponded to actin cables, green color-areas of low fluorescence corresponding *i.e.*, to remaining places containing actin and blue color-regions of cells without fluorescence signal. Different small letters (a,b,c,d,f) denote statistically significant differences (*p* < 0.05); (**B**) Mean length of microtubules bundles (MTs) in pixels expressed by the parameter of fiber length; (**C**) Mean width of microtubules bundles (MTs) in pixels expressed by the parameter of fiber width.

A particularly high accumulation of actin was observed in the vicinity of cell nuclei. Additionally, when cells were inoculated with *F. oxysporum* (+Si), bundles of actin were observed to thicken and fluorescence intensity increased in relation to +Sn cells. Quantitative analysis indicated a significant increase in the global density of microfilaments (MFs) in cells. This increase resulted mainly from an increase in occupancy regions of high fluorescence, which corresponded to actin cables (Figure 8A).

In non-inoculated cells of embryo axes cultured at a carbohydrate deficit (−Sn) a partial changes of the microfilaments meshwork was observed in relation to axes with an increased sugar level. Although the global density of MFs in the cell was not changed in comparison to +Sn, there was a slight reorganization of actin cytoskeleton: slightly increased occupancy of regions of high fluorescence. In turn, in inoculated cells with carbohydrate deficit (−Si) the greatest changes were observed in the actin cytoskeleton, *i.e.*, the length of all forms of microfilament bundles was reduced and the meshwork of microfilament bundles was fragmented. The global MF density was significantly decreased compared to all other variants (this may be a manifestation of depolymerization/fragmentation). This was done by a significant decrease in occupancy of regions of high intensity fluorescence corresponding to actin cables.

Tubulin cytoskeletons +Sn, +Si and −Sn cells of cortex in embryo axis (in horizontal section of cells) consisted of microtubules, which created bundles structure parallel to each other (Figure 7B). For the most part, these bundles were situated perpendicularly to the longer axis of the cell, and only occasionally at a small angle. In comparison to +Sn, in +Si and −Sn variants only slight changes were observed. Their structure was usually intact. Their reciprocal arrangement rarely changed. Therefore the cells, which were observed, had their microtubules bundles randomly scattered *i.e.*, within a cell there were regions, where bundles were more or less densely packed than in other regions. Moreover, the length of microtubules bundles in +Si and −Sn cells of embryo axes was a little shorter than in +Sn (Figure 8B). The width of microtubules bundles was not changed (Figure 8C). The tubulin cytoskeleton in −Si cells showed the most visible disorders *i.e.*, a decline or a considerable reduction of the number of microtubules, a significant shortening of length (fragmentation) of microtubules (Figure 8B), occurrence of big, irregularly shaped tubulin aggregates displaying strong fluorescence and the region with diffusive character of fluorescence (Figure 7B).

### 2.8. Discussion

This study demonstrates enhanced biosynthesis of genistein and other isoflavones, free aglycones by sucrose and monosaccharides (glucose and fructose) in *in vitro* cultured embryo axes of yellow lupine cv. Juno, resistant to fusariosis. As signal molecules, sugars strongly stimulated both the accumulation of genistein and other isoflavones (Figure 1) and the expression of the isoflavonoid biosynthetic genes (Figure 2). Infection with a hemibiotrophic fungus *F. oxysporum* significantly enhanced the synthesis of genistein, wighteone and luteone in cells of embryo axes of yellow lupine with high endogenous levels of sucrose, glucose and fructose (Figure 1A,C,D). A strong accumulation of genistein, particularly at later times post infection, was as a rule correlated with reduced levels of both sucrose and monosaccharides in tissues (Figure 3), which may indicate the involvement of these sugars as carbon skeletons in phenylpropanoid metabolism. Genistein as a free aglycone may be an inhibitor of pathogenic fungus *F. oxysporum*. 

A study by Shinde *et al.* [33] showed the effectiveness of sucrose for genistein and daidzein production, and growth when compared to glucose, fructose and maltose in suspension cultures of *Psoralea corylifolia*. The maximum production of genistein and daidzein was recorded when sucrose and maltose were used as sole sources of carbon. Suspension cell cultures enriched with sucrose (3%) stimulated accumulation of isoflavones, genistein and daidzein, when compared to glucose, fructose and maltose. Successive studies confirmed that sucrose in the medium is energetically the most advantageous source of carbon for cultivation of cell cultures of *P. corylifolia* and mainly for the biosynthesis of isoflavones. The productivity of daidzein was greater in hairy roots transformed by *Agrobacterium rhizogenes* than in untransformed roots [24]. Moreover, enhanced accumulation of isoflavone [34] and anthocyanin production [29,35,36] was also provided by controlled feeding of the carbon source in the growth medium. Increased accumulation of isoflavones by sucrose was also found in embryo axes of yellow lupine cv. Polo, being sensitive to fusariosis [9]. 

In turn, results recorded in this study are the first which show the effects of glucose and fructose on the mechanism regulating synthesis and accumulation of isoflavone free aglycones, including genistein, in the case of infection by *F. oxysporum*. Our other investigations revealed a strong accumulation of isoflavonoids, particularly free aglycones, in *F. oxysporum*-inoculated embryo axes of yellow lupine pretreated with nitric oxide (NO) and cultured with a high level of sucrose, to result from the amplification of the signal coming from sucrose, the nitric oxide donor and the pathogenic fungus [37]. The role of sugars as regulatory molecules is now generally recognized, as well as the dynamic nature of primary carbon metabolism (with efficient invertase-mediated conversion of sucrose to glucose and fructose) and complex interactions with hormone signaling pathways [30]. Moreover, results reported by Li *et al.* [29] suggest that the DELLA proteins can act as key positive regulators in the sucrose signaling pathway controlling anthocyanin biosynthesis and a point of integration of diverse metabolic and hormonal signals and growth. Moreover, enhanced post-infection accumulation of genistein, 2'hydroxygenistein, wighteone and luteone (prenylated isoflavones) was observed in leaves of lupine plants infected with *Colletotrichum lupini* spores [38]. A similar relationship was demonstrated by Muth *et al.* [39]. Synthesis of luteone and 2'hydroxygenistein was enhanced in the youngest leaves *L. angustifolius*, while that of wighteone in older leaves infected with a pathogenic fungus *C. lupini*, causing anthracnose.

As it was reported by Jeandet *et al.* [23], genetic manipulation of phytoalexins is investigated to increase disease resistance of plants. The first example of disease resistance resulting from foreign phytoalexin expression in a novel plant concerned a phytoalexin from grapevine, which was transferred to tobacco. Secondary metabolites characterized by antifungal activity can be an important line of defence against phytopathogenic fungi [8]. Plant antifungal metabolites present constitutively in healthy plants called phytoanticipins may be inhibitors of pathogenic fungi, or they may be synthesized *de novo* in response to their attack or other stress conditions (phytoalexins) [40]. These molecules may be used directly or be considered as precursors for the development of better fungicidal molecules. In addition, the same molecule may be a phytoalexin or a phytoanticipin in different organs of the same plant [41].

Results of investigations recorded within this and previous studies [9,42] showed a post-infection increase in the activity of β-glycosidase (Figure 4); however, it is not dependent on the level of carbohydrates in tissues. In embryo axes with high endogenous levels of sucrose, glucose and fructose (Figure 3), showing a considerable accumulation of isoflavones, including genistein (Figure 1), and a high level of expression of genes encoding enzymes of genistein biosynthesis (Figure 2), also a very strong generation of O_2_^•−^ was observed in their tissues (Figure 5). As early as 24 h and at the further time points post infection in embryo axes infected with *F. oxysporum* a very strong generation of this radical was found in cultures run on the medium with glucose, fructose and sucrose (Figure 5). Obviously, generation of O_2_^•−^ in these tissues is connected with the adopted defence strategy in infected embryo axes with a high carbohydrate level. During later stages after inoculation, lower generation of semiquinone radicals in +Si, +Gi and +Fi tissues in relation to −Si is probably connected with the strengthening and sealing of cell walls (Figure 6). 

In cells of embryo axes inoculated with *F. oxysporum* with a high endogenous level of sucrose (+Si), exhibiting high accumulation of genistein, actin bundles were observed to thicken and fluorescence intensity increased in relation to +Sn cells and other variants (Figure 7A). In turn, the length of microtubules bundles in +Si cells was a little shorter than in +Sn (Figure 8B).

Interestingly, the architecture of microfilaments and microtubules was preserved despite the inoculation, which may indicate that sucrose provides stabilization of the cytoskeleton. In turn, in inoculated cells with a carbohydrate deficit (−Si) the length of all forms of the actin cytoskeleton were reduced and fragmented. The global MF density was significantly decreased compared to all other variants which might suggest depolymerization/fragmentation [43]. Observations of the tubulin cytoskeleton in −Si also showed a decline or a considerable reduction of the number of microtubules, a significant shortening of length (fragmentation) of microtubules and the diffusive character of fluorescence (Figure 7B and Figure 8B). Hardham [44] reported that during the attack by fungal pathogens rapid morphological changes in the plant cytosol were observed that included the reorganization of the cortical microtubule array. Microtubule reorganization has been documented during both resistant and susceptible interactions. Nick [45] also reported that microtubules act as sensors and integrators for stimuli including pathogen attack. A mechanosensory function of microtubules, the cellular competence to induce defence genes in response to an elicitor correlates with microtubule stability. For example, the reduction of microtubule treadmilling to inhibit cell-to-cell transport of plant viruses. Disturbances of the microtubule structure were observed not only under the influence of biotic, but also abiotic stresses [46].

The regulation and turnover of the actin cytoskeleton requires concerted activities of hundreds of actin-binding proteins, which may respond to signals to polymerize or destroy actin filament networks. This constant formation and destruction of actin networks requires a huge expense of energy-on the order of millions of ATPs per second-and is thought to represent a surveillance mechanism to various biotic and abiotic stresses [47,48]. As it was reported by Henty-Ridilla *et al.* [49], the cytoskeleton plays a major role in organizing the structures, as well as responds rapidly to biotic stresses and supports numerous fundamental cellular processes, including vesicle trafficking, endocytosis and the spatial distribution of organelles and protein complexes. For years the actin cytoskeleton has been assumed to play a role in plant innate immunity against fungi and oomycetes (based on pharmacological studies). However, there is little evidence that the host-cell actin cytoskeleton participates in plant responses to phytopathogenic fungi. Actin microfilaments, on which targeted cytoplasm flow is dependent, may affect deposition of material outside the protoplast, which role during cell penetration by the attacking pathogen is to form papillae [50,51]. Studies of Henty-Ridilla *et al.* [49] revealed two distinct and statistically significant changes in actin filament organization following infection with a virulent pathogen, *i.e.*, an early and transient increase in actin filament density as well as a late increase in the extent of actin filament bundling. The requirement of the actin cytoskeleton for the activation of NADPH oxidase at the plasma membrane, the superoxide-generating system, as well as the Golgi complex, peroxisomes and endoplasmic reticulum trafficking toward sites of fungal and oomycete penetration, has been demonstrated mainly through pharmacological studies [52]. The reconfiguration and reorganization of the actin cytoskeleton as a result of pathogens in the human body cells is well-known [53], but in plant-host cells have more research is still required to elucidate this problem. It is important how this network is manipulated during infection by a diverse array of pathogens. Pathogenic microorganisms subvert normal-cell processes to create a specialized niche, which enhances their survival. A common and recurring target is the cytoskeleton, mainly the actin cytoskeleton, since the F-actin filaments are highly dynamic structures, which supramolecular organization is constantly modified to meet cellular needs. Actin dynamic behavior is regulated by a large number of binding proteins, which drive intracellular and extracellular signaling pathways [54].

Smertenko and Franklin-Tong [55] reported that the plant cytoskeleton is reorganised in response to programmed cell death (PCD), with remodelling of both microtubules and microfilaments taking place. In a majority of cases, the microtubule network depolymerises, remodelling of microfilaments can follow two scenarios, either being depolymerised and then forming stable foci, or forming distinct bundles and then depolymerising. Moreover, density of the filamentous actin (F-actin) network undergoes periodic alternating polymerisation and depolymerisation processes, while alterations of this periodicity may have a role in perceiving signals and generating responses. Moreover, cytoskeletal reorganization of the microtubules and microfilaments in *P. sylvestris* root cells after inoculation by *Heterobasidion annosum*, *Heterobasidion parviporum*, and *Heterobasidion abietinum* was noted also by Zadworny *et al.* [56]. 

Regardless of the above, in cells of non-inoculated axes of yellow lupine with a high endogenous level of sucrose (+Sn), the actin cytoskeleton was formed of long and thick actin cables surrounding the cells and their branches, creating a dense actin meshwork extending in various directions. A particularly high accumulation of actin in these cells was observed in the vicinity of cell nuclei. 

Wojtaszek *et al.* [57] reported that selective disruption of wall polysaccharides or covalent interactions between wall components leads to alterations in the organization of the actin cytoskeleton as well as disturbances of the mechanical stability of the whole cell. Short osmotic stress induces a rapid increase of F-actin at cross walls of maize root apices. Banaś *et al.* [58] reported that glucose and sucrose did not influence the pattern of the actin cytoskeleton in detached *Arabidopsis thaliana* leaves. In contrast, mannose caused the disappearance of filamentous structures and generated actin foci. Hexokinase as hexose sensor has been found to be associated with actin filaments in *Arabidopsis* [59]*.* Also another enzyme such as sucrose synthase, involved in sugar metabolism have been found to be associated with plant cytoskeleton [60]. Additionally, the organization of the actin cytoskeleton and vesicle trafficking may be modulated via another signal molecule such as NO in maize root apices [61].

## 3. Experimental Section

### 3.1. Materials

#### 3.1.1. Plant Material and Growth Conditions 

Experiments in this study were carried out on yellow lupine (*Lupinus luteus* L. cv. Juno). Cultivar Juno is resistant to fusariosis according to the data provided by the Plant Breeding Company in Poznań, Poland. Seeds were surface-sterilized, immersed in sterile water and left in an incubator (25 °C). After 6 h of imbibition, the seeds were transferred onto filter paper (in Petri dishes) and immersed in a small amount of water to support further absorption. After a subsequent 18 h, the seed coats were removed from the imbibed seeds and the cotyledons were removed to isolate the embryo axes. At the beginning of the experiment (time 0 h), the embryo axes were either inoculated with a *Fusarium oxysporum* f.sp. *lupini* spore suspension, or they were not inoculated. These were placed, within the next 20 min after cotyledon removal, in groups of four onto Whatman filter papers, which were subsequently transferred to sterile glass test tubes (diameter 3 cm, height 13.5 cm) containing 14 mL of Heller’s mineral medium [62]. There they were suspended in such a way that one end of the axis was immersed in the medium. A space was left below the paper to allow better aeration. After removal of the cotyledons, the embryo axes were dependent on the carbon source provided by the medium. Eight culture variants were applied: +S, embryo axes cultured *in vitro* on Heller’s medium supplemented with 60 mM sucrose [9,28,42,63,64], +G, 120 mM glucose, +F, 120 mM fructose and −S, embryo axes cultured *in vitro* on Heller’s medium without sucrose (−Sn, non-inoculated cultured without sucrose, −Si, inoculated cultured without sucrose). In addition, embryo axes before being transferred to sterile glass test tubes containing Heller’s mineral medium were not inoculated (the control, *i.e.*, +Sn, +Gn, +Fn) or inoculated with a pathogenic fungus *Fusarium oxysporum* f. sp. *lupini* (+Si, +Gi, +Fi).

The applied sucrose, glucose and fructose concentration was optimal to ensure appropriate growth of embryo axes, fresh and dry weight, as well as the uptake of minerals from the medium. Embryo axes were incubated in the dark at 25 °C. The model system was an equivalent of the stage in the development of germinating lupine seeds before the developing seedling emerges above the soil surface. At this heterotrophic stage, drastic changes may occur in the level of carbohydrates in the embryo axe of the germinating seed. The application of this model system was aimed at comparing two situations during heterotrophic stages of seed germination. The first stage is the stage when embryo axes are provided with an endogenous pool of soluble sugars due to the appropriately progressing mobilization of reserve nutrients in cotyledons, and the second stage is marked by a deficit of carbohydrates, so-called sugar starvation, resulting from disturbances in the mobilization of reserve substances. The experimental system, *i.e.*, embryo axes cultured *in vitro*, is a valuable model system, resembling natural growth condition. The applied experimental system provides a unique possibility to study the direct effect of sugars on plant defense responses to fungal infection. Activation of the phenylpropanoid pathway, *i.e.*, the level of expression of genes encoding PAL, CHS, CHI and IFS in the response of lupine embryo axes to a pathogenic fungus, was analyzed when an endogenous pool of soluble carbohydrates was provided by the exogenous sucrose, glucose, and fructose administration and during sugar starvation. Samples were collected for analyses at 0 h and after 24, 48, 72, and 96 h of culture, following that they were frozen at −80 °C to determine the contents of genistein and other isoflavones, carbohydrate concentrations, the relative expression level of genes encoding PAL, CHS, CHI and IFS using real-time PCR, free radical concentration using EPR and β-glucosidase activity. In order to determine actin and tubulin cytoskeletons, and the superoxide anion radical level fresh samples were collected for analyses at 0 h and after 24, 48, 72 and 96 h of culture. However, actin and tubulin cytoskeletons were detected only in embryo axes cultured on the medium either with sucrose or without it.

#### 3.1.2. Preparation of Spore Suspension and Inoculation

*Fusarium oxysporum* f. sp. *lupini* strain K-1018 (subsequently referred to as *F. oxysporum*) was obtained from the Collection of Plant Pathogenic Fungi, the Institute of Plant Protection, Poznań. The pathogen was incubated in the dark at 25 °C in Petri dishes (diameter 9 cm) on a potato dextrose agar (PDA) medium (pH 5.5, Sigma-Aldrich, Poznań, Poland). After 3 weeks of growth an *F. oxysporum* spore suspension was prepared. The spore suspension was obtained by washing the mycelium with sterile water and shaking with glass pearls. Then the number of spores was determined using a hemocytometer chamber (Bürker, Labart, Gdańsk, Poland). Embryo axes were inoculated with the spore suspension at a concentration of 5 × 10^6^ spores per 1 mL. Inoculation was performed by injecting 10 µL of spore suspension into the upper part of the embryo axis shoot and additionally also by spraying the upper part of the embryo axis shoot with the inoculum.

### 3.2. Methods

#### 3.2.1. Analysis of Isoflavonoids

##### 3.2.1.1. Isolation of Phenolic Compounds

Prior to LC profiling of isoflavone glucosides, frozen plant tissue was homogenized in 80% methanol (6 mL·g^−1^·FW) at 4 °C. After homogenization, 30 µL of luteolin standard (0.3 µmol) was added to each analyzed sample as an internal standard (LC retention time and UV spectral data did not interfere with those of the assayed compounds). Moreover, 2 mL of 80% methanol were also added to each sample. Samples were vortex-mixed and ultrasonic treatment was applied at room temperature for 30 min. Next, samples were centrifuged at 3000*× g* for 10 min. Supernatants obtained after centrifugation were evaporated at 45 °C. After evaporation of samples 1 mL of 80% methanol was added. Next samples were vortex-mixed, ultrasonic treatment was applied at room temperature for 15 min, and samples were centrifuged at 8000*× g* for 10 min.

##### 3.2.1.2. Liquid Chromatography (LC/UV/MS)

Quantitative analyses were performed on a Waters Acquity UPLC system (Manchester, UK), equipped with a diode array detector and a Poroshell 120 RP-18 column (100 mm × 2.1 mm, 2.7 µm; Agilent Technologies, Waldbronn, Germany). The UPLC system was additionally connected to an MS detector (micrOTOFq, Bruker Daltonics, Bremen, Germany) to provide proper identification of particular isoflavones based on MS and fragmentation spectra. Quantification of total isoflavones was achieved by integration of UV chromatograms at 259 nm and normalization to the peak of an internal standard (luteolin). The concentrations of lupine free aglycones, including genistein, were expressed in arbitrary units. During LC/UV/MS analyses the elution protocol was carried out using a 20 min gradient of two solvents: A (95% H_2_O, 4.5% acetonitrile, 0.5% formic acid, v/v/v) and B (95% acetonitrile, 4.5% H_2_O, 0.5% formic acid, v/v/v). Elution steps were as follows: 0–5 min gradient from 10% B to 30% B, isocratic to 12 min, 12–13 min linear gradient 95% B, 13–15 min isocratic at 95% of B. Free isoflavones (genistein, 2'-hydroxygenistein, wighteone, luteone) were identified by comparing their retention times with data obtained for standards. Genistein, 2'-hydroxygenistein, wighteone and luteone were run under identical chromatographic conditions. The above mentioned standards were obtained and characterized during earlier studies in our laboratory [65].

#### 3.2.2. Real-time RT-PCR

For the isolation of RNA, lupine embryo axes (500 mg) were frozen in liquid nitrogen and ground with a mortar and pestle in the presence of liquid nitrogen. Total RNA was isolated from 50 mg of tissue using the SV Total RNA Isolation System (Promega, Manheim, Germany), according to the supplier’s recommendations [28,37]. This protocol is adapted to processing of small tissue samples. The RNA level in samples was assayed spectrophotometrically at 260 nm. The A260/A280 ratio varied from 1.8 to 2.0 according to the manufacturer’s protocol. The transcript levels of target genes were analyzed by two-step quantitative RT-PCR (qRT-PCR). First-strand cDNA was synthesized from 1 µg of RNA using a High Capacity cDNA Reverse Transcription Kit (Applied Biosystems, Life Technologies Polska, Warszawa, Poland), according to the manufacturer’s protocol. qRT-PCR was performed using a 7900HT Fast Real-Time PCR System (Applied Biosystems) apparatus and the Power SYBR Green Master Mix kit (Applied Biosystems) in a final volume of 10 µL containing 2 µL of three-fold diluted cDNA or digested plasmid standard dilution and 2.5 pmol of each primer. Primers for amplification were designed on the basis of the cDNA sequences encoding genes of PAL, CHS, CHI, IFS and actin from yellow lupine (Table 1). In order to minimize inaccuracies due to genomic DNA contamination, amplicons were located in plausible joining regions of exons. In assays of CHI and IFS gene expression the applied thermal cycling conditions consisted of an initial denaturation at 95 °C for 10 min followed by 50 cycles at 95 °C for 15 s, 57 °C for 20 s and 60 °C for 1 min. In assays of CHS and actin gene expression the used program consisted of an initial denaturation at 95 °C for 10 min, followed by 50 cycles at 95 °C for 15 s, 52 °C for 20 s and 65 °C for 45 s. For the PAL gene expression assay thermal cycling conditions consisted of an initial denaturation at 95 °C for 10 min, followed by 45 cycles at 95 °C for 15 s, 56 °C for 15 s and 60 °C for 40 s. The quantification analysis was performed using the standard curve method. In each assay for a specific cDNA target standard curves were prepared using six 10-fold dilutions of the linear form of plasmid-cloned specific amplicons (pre-amplified PCR products) from 100 to 10,000,000 copies. Standards, cDNA samples and the no-template control were analyzed in three replications in each assay. The specificity of products was validated by dissociation curve analyses. The results were analyzed using the SDS 2.3 software (Applied Biosystems). The expression level of target genes was normalized to the actin expression value as a constitutively expressed reference gene.

**Table 1:** Real-time PCR primers used for quantification of mRNA levels of different genes.

Protein Name	Primer Name	Sequence (5'-3')	Amplicon (bp)
chalcone synthase	CHS F	ATCCTGATTTCTACTTCAGA	160
	CHS R	GGTGCCATATAAGCACAAA	
phenylalanine ammonia-lyase	PAL F	ATTTAACTCTGTACCATTGCCG	132
	PAL R	GGAGAACCAAACAGGGCG	
chalcone isomerase	CHI F	AGAATCAGCTGAGAAATGATA	207
	CHI R	GAGAAGGTTGTTAGACTTGT	
isoflavone synthase	IFS F	TGGGTTGTTGATGAGCTCTG	162
	IFS R	GTTTTTCTTGATACTTTGCTTG	
actin	Actin F	TGGTCGTCCTCGTCACACT	72
	Actin R	TGTGCCTCATCCCCAACATA	

#### 3.2.3. Extraction and Assay of β-glucosidase Activity

Frozen embryo axes (500 mg) were homogenized at 4 °C with a mortar and pestle in 3 mL of 0.05 M phosphate buffer (pH 7.0) containing 0.5% polyethylene glycol (PEG 6000). Polyclar AT (10 mg per 100 mg tissue) was added during extraction. Supernatants obtained after centrifugation (at 15,000*× g* for 20 min) were used to determine β-glucosidase (EC 3.2.1.21) activity. The activity was determined by the method of Nichols *et al.* [66]. The mixture containing 0.2 mL of extract, 0.2 mL 0.05 M phosphate buffer (pH 7.0) and 0.2 mL of 4-nitrophenyl-β-D-glucopyranoside as the substrate (2 mg·mL^−1^) was incubated for 1 h at 30 °C. After that time 0.6 mL of 0.2 N Na_2_CO_3_ was added. The formation of p-nitrophenol (p-NP) was measured at 400 nm (Perkin–Elmer Lambda 11 spectrophotometer, Norwalk, CT, USA).

#### 3.2.4. Electron Paramagnetic Resonance (EPR)

Samples of 800 mg fresh weight of embryo axes were frozen in liquid nitrogen and lyophilized in a Jouan LP3 freeze dryer (Saint-Herblain, France). The lyophilized material was transferred to EPR-type quartz tubes of 4 mm in diameter. Electron paramagnetic resonance measurements were performed at room temperature with a Bruker ELEXSYS X-band spectrometer (Rheinstettenstate, Germany). The EPR spectra were recorded as first derivatives of microwave absorption. A microwave power of 2 mW and a magnetic field modulation of about 2 G were used for all experiments to avoid signal saturation and deformation. EPR spectra of free radicals were recorded in the magnetic field range of 3,330–3,400 G and with 4096 data points. In order to determine the number of paramagnetic centers in the samples the spectra were double-integrated and compared with the intensity of the standard Al_2_O_3_:Cr^3+^ single crystal with a known spin concentration [37,64,67,68,69,70,71]. Before and after the first integration some background corrections of the spectra were made to obtain a reliable absorption signal before the second integration. These corrections were necessary due to the presence of small amounts of paramagnetic Mn^2+^ ions in the examined samples. Finally, EPR intensity data were calculated per 1 g of dry sample.

#### 3.2.5. Determination of Superoxide Anion Radical Content

Determination of superoxide anion radical (O_2_^•−^) content in biological samples was based on its ability to reduce nitro blue tetrazolium (NBT) [72] modified by Mai *et al.* [71]. Embryo axes (500 mg) were immersed in 10 mM potassium phosphate buffer (pH 7.8) containing 0.05% NBT and 10 mM NaN_3_ in a final volume of 3 mL and incubated for 1 h at room temperature. After incubation 2 ml of the reaction solution were heated at 85 °C for 15 min and rapidly cooled. The levels of O_2_^•−^ were expressed as absorbance at 580 nm per 1 g of fresh materials. The measurements were carried out in the Perkin Elmer Lambda 15 UV-Vis spectrophotometer.

#### 3.2.6. Carbohydrate Analysis

##### Extraction

Plant material (150 mg) was ground in liquid nitrogen using a 30 Hz laboratory ball mill (1 min, 2 balls per 2 mL Eppendorf tube) and flooded with 1.4 mL 80% cooled methanol (MeOH, HPLC). Next the samples were supplemented with 25 µL ribitol (1 mg/1 mL). Test tube contents were vortex-mixed in a thermomixer at 950 rpm for 10 min at room temperature, followed by centrifugation at 11.000*× g* for 10 min at 4 °C. The produced supernatant (250 µL) was transferred to Eppendorf tubes and evaporated in a speedvac at room temperature. 

##### Derivatisation

After sample desiccation in a dessicator each sample was supplemented with 50 µL methoxyamine (20 mg/mL in dry pyridine) and vortex-mixed in a thermomixer for 1.5 h at 37 °C, afterwards it was centrifuged for 10 s (short spin). Following centrifugation the samples were supplemented with 80 µL MSTF, again vortex-mixed in a thermomixer (30 min, 37 °C) and centrifuged at 11.000*× g* for 10 min. Prepared samples were transferred to inserts at 200 µL. 

##### GC-MS Analyses 

Endogenous carbohydrate levels were determined by gas chromatography coupled with mass spectrometry (GC-MS) (6890N gas chromatograph by Agilent with a GCT Premier mass spectrometer by Waters) using a DB-5MS column (30 m × 0.25 mm × 0.25 µm, J&W Scientific, Agilent Technologies, Palo Alto, CA, USA). Gradient: 70 °C for 2 min, followed by 10 °C/min up to 300 °C (10 min). Injector 250 °C, interface 250 °C, source 250 °C, *m/z* range: 50–650, EI+, electron energy 70 eV. Mass spectra were recorded in the electron ionization mode at a potential of 70 eV. Carbohydrate content was expressed in µg per 100 mg fresh matter. 

#### 3.2.7. Detection of Actin and Tubulin Cytoskeleton

In order to detect the actin cytoskeleton, material was collected from the tested experimental variants, *i.e.*, 48 h embryo axes of yellow lupine, both those non-inoculated and those inoculated with *F. oxysporum* and cultured on a medium with sucrose or without it. The cytoskeleton was detected using the fluorescence labelled phalloidin with the use of glycerol, applying the permeation method ([73] with modifications). Cross-sections were obtained from fragments of embryo axe shoots (fresh material), 3 mm below the inoculation site. Excised samples were incubated immediately in darkness for 3 h at 14–16 °C in the staining solution containing 0.02 µM Alexa Fluor^®^ 488-phalloidin (Invitrogen, Life Technologies Polska, Warszawa, Poland) and 3% (w/v) glycerol in microtubule-stabilizing buffer (MTBS: 50 mM PIPES, 5 mM EGTA and 5 mM MgSO_4_; pH 6.9). Next sections were rinsed several times and placed in MTSB buffer on a slide and examined under an LSM 510 confocal microscope (Zeiss, Jena, Germany) with fluorescence excitation with a light beam from a krypton-argon laser (488 nm). Emission of the fluorescent dye was recorded with a filter at 500–550 nm.

The tubulin cytoskeleton was detected in cells of embryo axes in yellow lupine by immunofluorescence ([74] with modifications). Plant material, *i.e.*, 3 mm fragments of shoots of yellow lupine embryo axes below the inoculation site were fixed in 4% PFA (paraformaldehyde) in MTSB buffer. Next the material was rinsed once with a mixture containing MTSB and PBS (phosphate buffered saline) (1:1) for 5 min, afterwards rinsed with PBS buffer (twice for 5 min each) at room temperature. Next, the material was dehydrated in a series of ethanol solution. In the last rinsing of the material in 100% ethanol 0.1% toluidine blue was added and then the material was embedded in Steedman’s wax. Samples were sectioned longitudinally into 12-µm-thick sections and mounted on glass slides coated with 0.1% poly-L-lysine solution. After the sections were dewaxed and rehydrated, they were incubated in 100 µL of diluted mouse monoclonal anti-α-tubulin (Sigma-Aldrich, Poznań, Poland) in PBS buffer (1:100) with 1% BSA and 5 mM sodium azide at 4 °C overnight in a humidity chamber. Samples were rinsed three times for 10 min each in PBS with 1% BSA and subsequently incubated with the secondary antibody, FITC-conjugated goat anti-mouse (Sigma) diluted in PBS (1:100), at 37 °C for 2 h in a dark humidity chamber. Samples were rinsed four times in PBS and mounted in Citifluor Antifadent AF1 (Citifluor Ltd., London, UK) under a glass coverslip. The materials were examined with an LSM 510 confocal microscope (Carl Zeiss). The samples were excited at 488 nm using a krypton-argon laser line and detected using a 505–550 nm bandpass filter.

To evaluate the microfilaments (MF) density, first the maximum intensity projections from the serial optical sections was obtained. The image was then colored by thresholding segmentation using. Red pixels were defined as areas of high intensity AlexaFluor fluorescence corresponded to the actin cables, green pixels represented areas of low intensity of fluorescence (corresponded, *i.e.*, to remaining places containing actin) and blue pixels regions without MF. Sum of red and green pixel areas represented the global amount of MF. Then were defined the AlexaFluor signal occupancy, which is an indicator of the proportion of pixel areas constituting the MFs of the total pixel areas constituting the cell region. The occupancy is defined by:

$$\text{Occupancy }(\text{\%})=100\;\frac{AreaMF}{AreaCell}$$

where *AreaMF* and *AreaCell* are the pixel areas constituting the MFs and cell region. The occupancy could evaluate the extent of MF depolymerization and fragmentation [43]. Projection feature of LSM510 software (Zeiss, Jena, Germany) was used for 3D image reconstruction of actin distribution in cells, while histogram display mode was used for the MF density in cells. 

In the case of microtubules analyzes the segmented images were converted into binary images. Thus obtained images were analyzed with software package KS300 3.0 (Carl Zeiss Vision, Germany). Individual MTs bundles were marked and the program then determined the following parameters: fiber length and fiber width (to characterize the morphology of MTs bundles). Fiber length and fiber width are defined by:

$$\text{Fiber length}=\frac{PerimeterF+\sqrt{PerimeterF^{2}-16\;AreaF}}{4}$$

$$\text{Fiber width}=\frac{PerimeterF-\sqrt{PerimeterF^{2}-16\;AreaF}}{4}$$

where *PerimeterF* and *AreaF* are the perimeter and area of the filled region, respectively. These parameters were measured in pixels. Calculations were made on the basis of three replicates of three plants each and at least 3–4 cells of each plant (about 30 cells in total). 

#### 3.2.8. Statistical Analysis

All determinations were performed in three independent experiments. Data shown are means of triplicates for each treatment; standard deviation was calculated and its range is shown in figures. The analysis of variance (ANOVA) was applied and results were compared in order to verify whether means from independent experiments within a given experimental variant were significantly different. Analysis of variance between treatment means was also carried out. The effects of two factors, *i.e.*, sugar (sucrose or glucose or fructose) and the pathogenic fungus (*F. oxysporum*), were investigated in the experiments.

Statistical significance of differences between average values for each pairs of indicators of physiological and biochemical analyses were carried out as appropriate elementary contrasts. Statistically significant differences (*p*-value) was set at *p* < 0.05 (Supplementary Materials). All calculations in the range of statistical analysis was performed using statistic packet Genstat 15.

## 4. Conclusions

In summary, this is the first study showing a stimulating effect of monosaccharides, glucose and fructose, on the mechanism regulating synthesis and accumulation of genistein and other isoflavones free aglycones, β-glucosidase activity, generation of free radicals in the case of infection by a pathogenic fungus. Furthermore, our findings are the first, which revealed the effect of infection on the organization of actin and tubulin cytoskeletons in terms of varied sucrose levels.

## Abbreviations

+Sn: embryo axes non-inoculated and cultured *in vitro* on Heller’s medium supplemented with 60 mM sucrose
+Gn: embryo axes non-inoculated and cultured *in vitro* on Heller’s medium supplemented with 120 mM glucose
+Fn: embryo axes non-inoculated and cultured *in vitro* on Heller’s medium supplemented with 120 mM fructose
−Sn: non-inoculated cultured without sucrose
+Si: inoculated and cultured with 60 mM sucrose
+Gi: inoculated and cultured with 120 mM glucose
+Fi: inoculated and cultured with 120 mM fructose
−Si: inoculated and cultured without sucrose
CHI: chalcone isomerase
CHS: chalcone synthase
EPR: electron paramagnetic resonance
IFS: isoflavone synthase
PAL: phenylalanine ammonia-lyase

## Supplementary Materials

Supplementary materials can be accessed at: http://www.mdpi.com/1420-3049/19/9/13392/s1.

## Supplementary Files

Supplementary File 1
