# Supplementary material for: Effects of Endogenous Signals and Fusarium oxysporum on the Mechanism Regulating Genistein Synthesis and Accumulation in Yellow Lupine and Their Impact on Plant Cell Cytoskeleton
Source: Molecules. 2014 Aug 29;19(9):13392–421. doi: 10.3390/molecules190913392 (PMC6271453; doi:10.3390/molecules190913392)
Supplement: Supplementary File 1 [file molecules-19-13392-s001.pdf]

Supplementary Materials

Statistical significance of differences between the average values of each pairs for accumulation of genistein and other isoflavones. Statistically significant differences (p-value) was assumed at $p < 0.05$.

Genistein

Time (h)	Contrast	Value of Contrast	*p*-Value	Time (h)	Value of Contrast	*p*-Value
**0**	0n:0i	−0.0473	0.033			
	+Sn:+Si	0.1033	0.101		−0.1270	0.106
	+Gn:+Gi	−0.2893	0.010		−0.2417	0.012
	+Fn:+Fi	0.0486	0.395		−0.6283	<0.001
**24**	−Sn:−Si	−0.0706	0.047	**72**	−0.2017	0.009
	+Si:−Si	0.0330	0.289		0.2020	0.026
	+Gi:−Si	0.2640	0.014		0.1110	0.150
	+Fi:−Si	0.00006	0.999		0.4007	0.002
	+Sn:+Si	−0.1833	0.031		−1.478	<0.001
	+Gn:+Gi	−0.3913	<0.001		−0.9520	<0.001
	+Fn:+Fi	−0.2333	0.029		−1.103	0.019
**48**	−Sn:−Si	−0.6420	<0.001	**96**	−0.1153	0.172
	+Si:−Si	−0.4627	0.005		1.490	<0.001
	+Gi:−Si	−0.1460	0.086		0.8577	0.002
	+Fi:−Si	−0.3877	0.014		0.9517	0.004

2'-Hydroxygenistein

Time (h)	Contrast	Value of Contrast	*p*-Value	Time (h)	Value of Contrast	*p*-Value
**0**	0n:0i	0.0533	0.022			
	+Sn:+Si	0.0120	0.840		0.9657	<0.001
	+Gn:+Gi	−0.2523	<0.001		1.749	<0.001
	+Fn:+Fi	0.3080	<0.001		1.282	<0.001
**24**	−Sn:−Si	−0.0663	0.163	**72**	0.0133	0.106
	+Si:−Si	0.1370	0.049		0.2887	<0.001
	+Gi:−Si	0.2147	0.006		0.1607	0.044
	+Fi:−Si	−0.0043	0.924		0.0433	0.034
	+Sn:+Si	1.165	<0.001		1.543	<0.001
	+Gn:+Gi	1.325	0.007		0.5640	<0.001
	+Fn:+Fi	1.219	0.003		0.9840	<0.001
**48**	−Sn:−Si	0.0833	<0.001	**96**	0.0986	<0.001
	+Si:−Si	0.2460	0.013		0.2210	0.013
	+Gi:−Si	0.1010	<0.001		0.2590	<0.001
	+Fi:−Si	0.0860	<0.001		0.1540	0.031

Wighteon

Time (h)	Contrast	Value of Contrast	*p*-Value	Time (h)	Value of Contrast	*p*-Value
**0**	0n:0i	−0.0272	0.038			
	+Sn:+Si	−0.3823	<0.001		1.121	<0.001
	+Gn:+Gi	−0.2583	<0.001		1.255	<0.001
	+Fn:+Fi	−0.4880	<0.001		−0.4727	<0.001
**24**	-Sn:−Si	−0.2910	<0.001	**72**	0.0103	0.691
	+Si:−Si	0.1947	0.002		0.5460	<0.001
	+Gi:−Si	−0.0736	0.004		0.5170	<0.001
	+Fi:−Si	0.2277	0.018		0.7720	<0.001
	+Sn:+Si	−0.7563	<0.001		0.7067	<0.001
	+Gn:+Gi	−1.298	<0.001		1.684	<0.001
	+Fn:+Fi	−0.0066	0.678		1.315	<0.001
**48**	−Sn:−Si	0.0413	0.419	**96**	0.1980	0.006
	+Si:−Si	0.7147	<0.001		0.3933	0.003
	+Gi:−Si	1.323	<0.001		0.2870	0.001
	+Fi:−Si	0.3567	<0.001		0.1707	0.002

Luteon

Time (h)	Contrast	Value of Contrast	*p*-Value	Time (h)	Value of Contrast	*p*-Value
**0**	0n:0i	0.0310	0.041			
	+Sn:+Si	−0.2467	<0.001		−0.4324	<0.001
	+Gn:+Gi	−0.5930	<0.001		−0.5050	0.001
	+Fn:+Fi	−0.4550	<0.001		−1.446	<0.001
**24**	−Sn:−Si	−0.3887	<0.001	**72**	−0.4323	<0.001
	+Si:−Si	0.0996	0.009		0.4427	0.002
	+Gi:−Si	0.1103	0.007		0.6590	<0.001
	+Fi:−Si	0.1303	0.010		1.126	<0.001
	+Sn:+Si	−0.8723	<0.001		−1.290	<0.001
	+Gn:+Gi	−1.701	<0.001		−0.6073	<0.001
	+Fn:+Fi	−0.5290	<0.001		−0.2763	0.001
**48**	−Sn:−Si	−0.2123	0.001	**96**	0.5250	<0.001
	+Si:−Si	0.7347	<0.001		1.794	<0.001
	+Gi:−Si	1.441	<0.001		1.139	<0.001
	+Fi:−Si	0.4437	<0.001		0.8910	<0.001

Statistical significance of differences between the average values of each pairs for expression levels of isoflavone biosynthetic pathway genes. Statistically significant differences (*p*-value) was assumed at $p < 0.05$.

PAL

Time (h)	Contrast	Value of Contrast	*p*-Value	Time (h)	Value of Contrast	*p*-Value
**0**	0n:0i	−0.064	0.108			
**24**	+Sn:+Si	0.1590	0.028	**72**	−1.475	<0.001
	+Gn:+Gi	−0.3607	<0.001		−1.552	<0.001
	+Fn:+Fi	−0.1913	0.001		−0.786	<0.001
	−Sn:−Si	−0.9157	<0.001		−2.231	<0.001
	+Si:−Si	−0.4617	<0.001		−0.694	<0.001
	+Gi:−Si	0.0106	0.757		−0.354	<0.001
	+Fi:−Si	−0.3613	<0.001		−1.249	<0.001
**48**	+Sn:+Si	−0.6617	<0.001	**96**	−1.869	<0.001
	+Gn:+Gi	−0.6737	<0.001		−0.824	<0.001
	+Fn:+Fi	−0.3940	<0.001		−4.280	<0.001
	−Sn:−Si	−0.6370	<0.001		0.517	0.002
	+Si:−Si	0.3623	<0.001		2.561	<0.001
	+Gi:−Si	0.4097	<0.001		2.209	0.002
	+Fi:−Si	0.2067	0.004		5.033	<0.001

CHS

Time (h)	Contrast	Value of Contrast	*p*-Value	Time (h)	Value of Contrast	*p*-Value
**0**	0n:0i	−1.116	<0.001			
**24**	+Sn:+Si	−1.652	<0.001	**72**	−4.289	<0.001
	+Gn:+Gi	−2.040	<0.001		−3.911	<0.001
	+Fn:+Fi	−2.240	<0.001		−3.029	<0.001
	−Sn:−Si	−2.549	<0.001		−8.179	<0.001
	+Si:−Si	0.068	0.699		−3.405	<0.001
	+Gi:−Si	0.339	0.168		−3.497	<0.001
	+Fi:−Si	0.325	0.198		−4.761	<0.001
**48**	+Sn:+Si	−2.176	<0.001	**96**	−7.276	<0.001
	+Gn:+Gi	−2.202	<0.001		−4.210	<0.001
	+Fn:+Fi	−2.953	<0.001		−8.054	<0.001
	−Sn:−Si	−2.985	<0.001		0.129	0.004
	+Si:−Si	0.367	0.027		7.798	<0.001
	+Gi:−Si	−0.150	0.241		5.290	<0.001
	+Fi:−Si	0.584	0.010		8.679	<0.001

CHI

Time (h)	Contrast	Value of Contrast	*p*-Value	Time (h)	Value of Contrast	*p*-Value
**0**	0n:0i	−0.188	0.003			
	+Sn:+Si	−0.580	0.040		−1.024	<0.001
	+Gn:+Gi	−0.536	0.006		−1.266	<0.001
	+Fn:+Fi	−0.574	0.002		−0.568	0.004
**24**	−Sn:−Si	−0.962	<0.001	**72**	−1.572	<0.001
	+Si:−Si	−0.070	0.612		−0.231	0.126
	+Gi:−Si	−0.007	0.948		0.001	0.995
	+Fi:−Si	−0.026	0.614		−0.654	0.003
	+Sn:+Si	−0.406	0.004		−1.328	<0.001
	+Gn:+Gi	−0.650	0.009		−0.717	0.001
	+Fn:+Fi	−1.124	<0.001		−2.276	<0.001
**48**	−Sn:−Si	−0.688	<0.001	**96**	0.629	0.011
	+Si:−Si	0.362	0.013		2.179	<0.001
	+Gi:−Si	0.306	0.008		1.889	<0.001
	+Fi:−Si	0.574	<0.001		3.328	<0.001

IFS

Time (h)	Contrast	Value of Contrast	*p*-Value	Time (h)	Value of Contrast	*p*-Value
**0**	0n:0i	1.916	<0.001			
	+Sn:+Si	−1.087	<0.001		−2.685	<0.001
	+Gn:+Gi	−1.706	<0.001		−3.310	<0.001
	+Fn:+Fi	−1.483	<0.001		−1.539	<0.001
**24**	-Sn:−Si	−1.897	<0.001	**72**	−4.512	<0.001
	+Si:−Si	−0.110	0.419		−1.314	0.004
	+Gi:−Si	0.372	0.035		−0.522	0.050
	+Fi:−Si	−0.001	0.993		−2.381	<0.001
	+Sn:+Si	−1.102	<0.001		−4.453	<0.001
	+Gn:+Gi	−1.772	<0.001		−2.625	<0.001
	+Fn:+Fi	−1.806	<0.001		−5.880	<0.001
**48**	-Sn:−Si	−1.984	<0.001	**96**	0.117	0.006
	+Si:−Si	0.002	0.981		5.025	<0.001
	+Gi:−Si	0.284	0.058		3.757	0.002
	+Fi:−Si	0.255	0.020		6.755	<0.001

Statistical significance of differences between the average values of each pairs for endogenous level of sugars. Statistically significant differences (*p*-value) was assumed at $p < 0.05$.

Sucrose

Time (h)	Contrast	Value of Contrast	*p*-Value	Time (h)	Value of Contrast	*p*-Value
**0**	0n:0i	−651.0	0.020			
	+Sn:+Si	−84.47	0.781		2010	0.010
	+Gn:+Gi	802.3	0.005		2123	<0.001
	+Fn:+Fi	90.20	0.556		1635	<0.001
**24**	−Sn:−Si	272.5	0.015	**72**	53.10	0.008
	+Si:−Si	1385	0.006		398.4	<0.001
	+Gi:−Si	962.4	0.002		278.7	0.002
	+Fi:−Si	1527	<0.001		457.6	<0.001
	+Sn:+Si	607.7	0.005		2141	0.009
	+Gn:+Gi	176	0.359		1255	0.015
	+Fn:+Fi	202.6	0.112		1761	<0.001
**48**	−Sn:−Si	216.9	0.007	**96**	10.07	0.562
	+Si:−Si	1699	0.003		242.9	0.017
	+Gi:−Si	1316	0.003		28.27	<0.001
	+Fi:−Si	1862	<0.001		12.57	0.008

Glucose

Time (h)	Contrast	Value of Contrast	*p*-Value	Time (h)	Value of Contrast	*p*-Value
**0**	0n:0i	−53.53	<0.001			
	+Sn:+Si	−37.73	0.208		1668	0.007
	+Gn:+Gi	428.3	0.012		842.2	0.010
	+Fn:+Fi	77.27	0.104		1284	0.023
**24**	-Sn:−Si	−5.833	0.468	**72**	15.70	<0.001
	+Si:−Si	200.8	0.001		309.1	0.002
	+Gi:−Si	328.0	0.014		644.9	0.016
	+Fi:−Si	203.1	<0.001		168.6	0.002
	+Sn:+Si	196.0	0.011		1380	<0.001
	+Gn:+Gi	10.27	0.845		502.8	0.021
	+Fn:+Fi	−351.5	0.029		1659	0.013
**48**	−Sn:−Si	−2.133	0.170	**96**	16.50	0.003
	+Si:−Si	498.7	0.003		160.7	0.014
	+Gi:−Si	542.3	0.005		912.7	<0.001
	+Fi:−Si	799.4	0.015		62.90	<0.001

Fructose

Time (h)	Contrast	Value of Contrast	*p*-Value	Time (h)	Value of Contrast	*p*-Value
**0**	0n:0i	−391.4	0.003			
	+Sn:+Si	−626.5	0.006		2566	0.003
	+Gn:+Gi	953.1	<0.001		2078	0.020
	+Fn:+Fi	1509	<0.001		1703	0.001
**24**	−Sn:−Si	164.5	0.020	**72**	11.43	0.046
	+Si:−Si	1092	<0.001		981.5	0.008
	+Gi:−Si	387.9	0.003		347.1	0.015
	+Fi:−Si	−14.53	0.514		1654	<0.001
	+Sn:+Si	256.8	0.002		2417	<0.001
	+Gn:+Gi	−210.4	0.218		2422	0.017
	+Fn:+Fi	−915.0	0.001		−1804	0.011
**48**	-Sn:−Si	20.60	0.005	**96**	18.07	<0.001
	+Si:−Si	1665	<0.001		247.7	0.016
	+Gi:−Si	1087	0.011		29.37	<0.001
	+Fi:−Si	2662	0.001		4688	0.003

Statistical significance of differences between the average values of each pairs for β-glucosidase activity. Statistically significant differences (*p*-value) was assumed at $p < 0.05$.

β-Glucosidase

Time (h)	Contrast	Value of Contrast	*p*-Value	Time (h)	Value of Contrast	*p*-Value
**0**	0n:0i	−0.1550	0.049			
	+Sn:+Si	0.2533	0.180		−1.393	0.177
	+Gn:+Gi	−0.4433	0.201		−4.530	0.003
	+Fn:+Fi	0.7667	0.090		−1.773	0.047
**24**	−Sn:−Si	−2.400	0.008	**72**	−8.940	<0.001
	+Si:−Si	0.9267	0.060		−3.770	0.016
	+Gi:−Si	−0.4800	0.315		−2.087	0.088
	+Fi:−Si	−0.7633	0.170		−7.280	<0.001
	+Sn:+Si	−1.350	0.021		−7.613	<0.001
	+Gn:+Gi	−2.113	0.008		−4.163	0.009
	+Fn:+Fi	−1.217	0.155		−2.823	0.036
**48**	-Sn:−Si	−2.800	0.001	**96**	−10.44	<0.001
	+Si:−Si	−2.140	0.006		−3.617	0.007
	+Gi:−Si	−1.577	0.017		−7.020	<0.001
	+Fi:−Si	−2.577	0.009		−9.230	<0.001

Statistical significance of differences between the average values of each pairs for superoxide anion radical generation. Statistically significant differences (*p*-value) was assumed at $p < 0.05$.

Superoxide Anion Radical

Time (h)	Contrast	Value of Contrast	*p*-Value	Time (h)	Value of Contrast	*p*-Value
**0**	0n:0i	1.550	<0.001			
	+Sn:+Si	−2.500	<0.001		−3.200	<0.001
	+Gn:+Gi	−4.300	<0.001		−2.133	<0.001
	+Fn:+Fi	−0.100	0.468		−4.883	<0.001
**24**	−Sn:−Si	−1.300	0.002	**72**	−4.950	<0.001
	+Si:−Si	2.700	<0.001		−1.450	0.002
	+Gi:−Si	3.733	<0.001		−1.250	0.003
	+Fi:−Si	0.700	0.009		0.5000	0.041
	+Sn:+Si	−3.750	<0.001		−2.967	<0.001
	+Gn:+Gi	−4.467	<0.001		−2.217	<0.001
	+Fn:+Fi	−5.100	<0.001		−2.350	<0.001
**48**	−Sn:−Si	−4.750	<0.001	**96**	−2.750	<0.001
	+Si:−Si	−0.5000	0.041		0.5667	0.038
	+Gi:−Si	−0.1833	0.212		−0.4333	0.050
	+Fi:−Si	0.4167	0.048		−0.2000	0.335

Statistical significance of differences between the average values of each pairs for semiquinone radical concentration. Statistically significant differences (*p*-value) was assumed at $p < 0.05$.

Concentration of Free Radicals

Time (h)	Contrast	Value of Contrast	*p*-Value	Time (h)	Value of Contrast	*p*-Wartość
**0**	0n:0i	0.025				
	+Sn:+Si	−0.3867	0.006		−1.310	<0.001
	+Gn:+Gi	−0.3867	<0.001		−1.660	<0.001
	+Fn:+Fi	−0.2067	0.232		−1.057	<0.001
**24**	−Sn:−Si	0.0866	0.427	**72**	−2.437	0.021
	+Si:−Si	0.1167	0.354		−0.5600	0.263
	+Gi:−Si	0.2767	0.051		−0.0566	0.890
	+Fi:−Si	0.1967	0.315		−0.8900	0.133
	+Sn:+Si	−0.1000	0.621		−3.507	0.012
	+Gn:+Gi	−0.0333	0.653		−2.744	<0.001
	+Fn:+Fi	−0.0200	0.895		−2.490	0.006
**48**	−Sn:−Si	−0.7000	0.051	**96**	−5.950	0.010
	+Si:−Si	−0.1300	0.623		−2.027	0.051
	+Gi:−Si	−0.1900	0.369		2.620	0.014
	+Fi:−Si	−0.4333	0.111		−3.023	0.017
